# Evaluation of Diagnostic Performance and Inter-Reader Agreement of Prostate Imaging After Focal Ablation (PI-FAB) on Post-Focal Therapy (FT) Magnetic Resonance Imaging (MRI)

**DOI:** 10.3390/curroncol33070417

**Published:** 2026-07-11

**Authors:** Guanqi Hang, Zhuyi Rebekah Lee, Anna Lois Lai, Jyothirmayi Velaga, Hua Thun Ho, Shelby Xuan Lin Lam, Yu Guang Tan, Nye Thane Ngo, John Shyi P. Yuen, Li Yan Khor, Melvin Lee Kiang Chua, Kae Jack Tay, Yan Mee Law

**Affiliations:** 1 Department of Cardiothoracic and Abdominal Radiology, Singapore General Hospital, Singapore 169608, Singaporelaw.yan.mee@singhealth.com.sg (Y.M.L.); 2 Department of Medical Imaging, St. Vincent’s Hospital, Sydney 2010, Australia; jyothirmayi.velaga@svha.org.au; 3 Department of Urology, Singapore General Hospital, Singapore 169608, Singaporetay.kae.jack@singhealth.com.sg (K.J.T.); 4 Department of Anatomical Pathology, Singapore General Hospital, Singapore 169608, Singapore; 5 Divisions of Radiation Oncology and Medical Sciences, National Cancer Centre Singapore, Singapore 168583, Singapore

**Keywords:** prostate cancer, mpMRI, focal therapy, cryotherapy, irreversible electroporation, PI-FAB

## Abstract

Focal therapy (FT) for localized prostate cancer induces architectural changes that complicate post-treatment multiparametric magnetic resonance imaging (mpMRI) surveillance. The Prostate Imaging after Focal Ablation (PI-FAB) system was developed to standardize the evaluation of in-field recurrence on mpMRI. This study assessed the diagnostic performance and inter-reader agreement of PI-FAB following focal cryoablation for localized prostate cancer. In this retrospective study (October 2019 to January 2024), 85 patients (140 lesion sites) underwent post-FT mpMRI and biopsy. Two radiologists (11 and 4 years of experience) independently scored mpMRIs using the three-point PI-FAB scale. Metrics included sensitivity, specificity, PPV, NPV, and accuracy. Inter-reader agreement was measured via quadratic weighted Cohen’s kappa (κ). Reader 1 (more experienced) demonstrated 83.9% sensitivity, 84.4% specificity, 60.5% PPV, and 94.8% NPV. Reader 2 demonstrated 71.4% sensitivity, 87.5% specificity, 58.8% PPV, and 92.5% NPV. Both achieved 84.3% accuracy. Inter-reader agreement was moderate (κ = 0.60). PI-FAB provides good diagnostic performance for detecting in-field recurrence after cryoablation. While reader experience may impact sensitivity, moderate agreement across experience levels supports PI-FAB’s validity and potential clinical utility in standardizing post-FT surveillance.

## Simple Summary

Our work evaluates a recent MRI scoring system (Prostate Imaging after Focal Ablation, PI-FAB) designed to detect recurrent prostate cancer after focal therapy. Focal therapy targets only cancer in the prostate without treatment to the whole prostate, performed for patients with intermediate risk prostate cancer. Monitoring patients after focal therapy is challenging because the prostate tissue becomes distorted and difficult to interpret on MRI. Our team reviewed follow-up MRI scans and biopsy results from men after focal treatment. Two radiologists with different levels of experience independently used PI-FAB to detect cancer recurrence. Overall, PI-FAB performed well in reliably identifying cancer recurrence. The more experienced radiologist achieved slightly better results, suggesting that specialist experience improves interpretation accuracy. The study concludes that PI-FAB is a promising and practical tool for standardizing MRI surveillance after focal prostate cancer therapy, potentially improving consistency in follow-up care and detecting recurrent disease more reliably.

## 1. Introduction

Prostate cancer is currently the most prevalent solid-organ malignancy among men in the developed world, with approximately 1.3 million new cases diagnosed annually worldwide [1,2]. Advances in diagnostic and therapeutic modalities have improved outcomes but the challenge of balancing effective treatment with the preservation of quality of life persists [2].

Focal therapy (FT) involves ablating the clinically significant prostate cancer while conserving critical adjacent structures such as the neurovascular bundle [3,4]. This approach potentially reduces the adverse effects associated with radical treatments, including erectile dysfunction, urinary incontinence, and proctitis, thereby enhancing functional outcomes while maintaining effective oncological control [4,5,6]. In our institution, cryoablation and irreversible electroporation (IRE) have become preferred options for limited-volume clinically significant prostate cancer (csPCA), balancing oncologic control with functional preservation [1,3,6].

Accurately detecting cancer recurrence after FT remains a major diagnostic hurdle. While cancer in untreated prostate tissue (out-field recurrence) may still be identified by multiparametric magnetic resonance imaging (mpMRI) using the standard Prostate Imaging: Reporting and Data System (PI-RADS v2.1) [7], there is reduced accuracy in the ablation zone [8]. Recurrence within the ablation site (in-field recurrence) is often confounded by treatment-induced changes of fibrosis, edema, and necrosis, which can obscure or mimic recurrent disease [9,10]. Subjective interpretation of in-field post-FT mpMRI changes may lead to unnecessary biopsies or delayed detection of true recurrences. This underscores the necessity of standardized scoring systems for post-FT mpMRI assessment.

The Prostate Imaging after Focal Ablation (PI-FAB) score is a three-point scale that stratifies patients based on the likelihood of in-field csPCA recurrence amid treatment-related artifacts [11]. If routinely adopted, PI-FAB has the potential to optimize post-FT mpMRI interpretation, enhance multidisciplinary communication of results, inform clinical decision-making, and facilitate multicenter research. However, its performance in real-world settings among readers with different levels of expertise must be first evaluated and validated [11,12].

Our study aims to evaluate the diagnostic accuracy and inter-reader agreement of the PI-FAB score in detecting biopsy-proven in-field csPCA recurrences following focal cryoablation or IRE among two readers of differing experience, providing critical insights into its clinical performance.

## 2. Materials and Methods

### 2.1. Patient Population

This retrospective study included consecutive patients who underwent primary FT for intermediate risk localized csPCA at a single institution from 2019 to 2024. The patients were recruited in a single-arm Phase II prospective clinical trial from 2019 to 2021 and subsequently in a prospective observational cohort extension from 2021 to 2023, with identical patient selection, treatment, and surveillance protocols, as previously described [13]. The study was approved by our institutional review board (IRB). Written informed consent was obtained from all participants.

At 12 months post-FT, patients underwent mandatory mpMRI and transperineal MRI-TRUS fusion targeted and systematic prostate re-biopsy, comprising (1) targeted biopsy over the ablation sites, (2) targeted biopsy of PI-RADS v2.1 category ≥ 3 lesions detected on post-treatment mpMRI in the untreated gland, and (3) systematic saturation biopsy of the remaining prostate. All patients underwent primary focal cryotherapy or IRE. After the first post-FT mpMRI, patients underwent routine surveillance mpMRI at 3 and 5 years. Biopsy was performed on a for-cause basis 3 and 5 years post-FT. All prostate biopsies were performed by a single expert urologist (K.J.T) with 10 years of experience in performing transperineal magnetic resonance imaging–transrectal ultrasound (MRI-TRUS) fusion biopsy (Biobot Surgical, Singapore).

**Figure 1:**
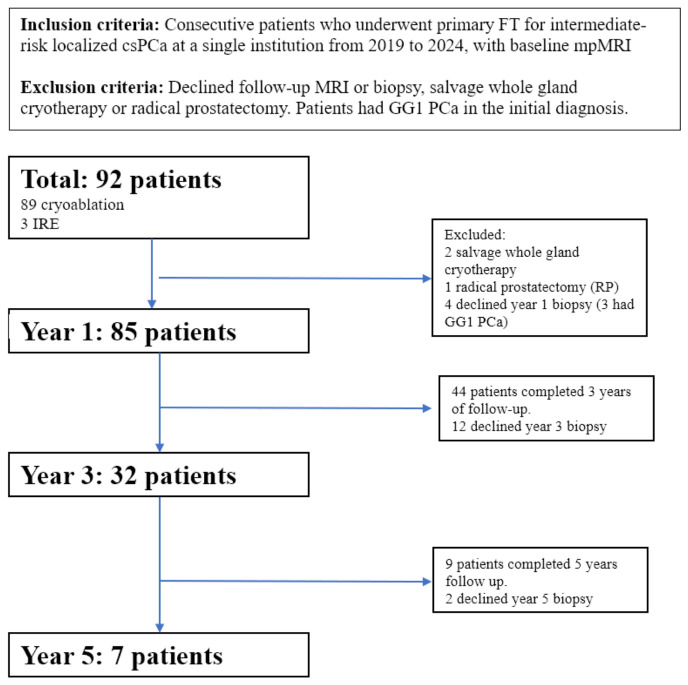
**Flowchart illustrating the patient selection process at different follow-up time points.** Years listed are duration after focal therapy for localized prostate cancer.

Only patients with available post-FT mpMRI and histopathological results of targeted biopsies at the ablation sites were included in the analysis. Patients who declined biopsy or mpMRI evaluation were excluded (Figure 1). The histopathological results of targeted biopsies at ablation sites served as the reference standard. A lesion with ISUP/WHO grade group ≥ 2 was classified as clinically significant in-field recurrence (csPCA).

At 1 year post-treatment, follow-up multiparametric MRI (mpMRI) with confirmatory biopsy was conducted for 85 men. Seven patients were excluded from the 1-year analysis due to receiving salvage whole-gland cryotherapy (*n* = 2), refusing biopsy (*n* = 4), or having an interval radical prostatectomy (*n* = 1). At 3 years post-treatment, 44 men remained in follow-up; however, biopsy data were available for 32 men, as 12 patients who underwent 3-year mpMRI declined biopsy. At 5 years post-treatment, follow-up mpMRI with biopsy was available for 7 men, mainly due to insufficient follow-up duration (<5 years) and biopsy refusal (*n* = 2). Over the 5-year follow-up, a total of 140 lesion sites were identified and included in the analysis.

### 2.2. MRI Data Acquisition

Patients underwent high-field mpMRI on a 3-Tesla MRI (Magnetom Skyra or Magnetom Vida; Siemens, Erlangen, Germany) with a 60-channel pelvic (surface) phased array coil without endorectal coil. The image acquisition protocols include T1W (T1 weighted imaging), T2W (T2 weighted imaging), ADC (apparent diffusion coefficient) maps, and DWI. For DWI, all 4 B-values (0–50, 500, 1000, 1800 s/mm^2^) were acquired, and ADC mapping was generated with all available B-values. A dynamic contrast-enhanced (DCE) sequence was obtained using gadoterate meglumine (DOTAREM, Guerbet KKC, Bloomington, IN, USA) intravenous injection at a dose of 0.1 mmol/kg body weight at a rate of 2–3 mL/sec, followed by a 20 mL saline flush. Imaging parameters were previously described [14].

### 2.3. Image Interpretation

The mpMRI images were reviewed on a PACS workstation (Carestream, Rochester, NY, USA) by two genitourinary radiologists with different levels of experience (Y.M.L. with 11 years of experience and 1000 prostate MRI scans annually, and A.L.L. with 4 years of experience and 500 prostate MRI scans annually) independently, using the PI-FAB scoring system as proposed by Giganti et al. [11]. All scans were anonymized. The radiologists were provided only with patient age, ethnicity, serum prostate specific antigen (PSA) level, sites, and the date of the FT. Readers were blinded to histopathological results.

### 2.4. PI-FAB Scoring

PI-FAB is a 3-point scoring system, used to assess cancer recurrence at the site of FT (PI-FAB 1 = no recurrence; PI-FAB 2 = equivocal; PI-FAB 3 = suspicious for recurrence) [11]. The PI-FAB system was developed by Giganti et al. based on the recognition that standard PI-RADS v2.1 criteria were not designed for post-ablation tissue and that treatment-induced changes—including fibrosis, hemosiderin deposition, edema, and necrosis—can closely mimic or obscure recurrent tumor on mpMRI [11]. PI-FAB scores are assigned after holistic assessment of all three mpMRI sequences, with each score reflecting the overall likelihood of in-field clinically significant recurrence: a PI-FAB 1 designation indicates imaging features consistent with expected post-treatment change and no evidence of recurrence; PI-FAB 2 indicates equivocal findings that may represent either residual/recurrent disease or benign post-treatment sequelae, warranting clinical correlation; and PI-FAB 3 indicates features highly suspicious for in-field csPCA recurrence, such as nodular early arterial enhancement on DCE, restricted diffusion on high b-value DWI with corresponding low ADC values, and focal T2 hypointensity distinct from background ablation changes [11].

PI-FAB assesses the three MRI sequences in sequential order: (1) dynamic contrast enhancement (DCE) (dominant sequence); (2) DWI high-b-value and ADC Map; and (3) T2-WI sequence with DCE MRI as the dominant sequence.

**Figure 2:**
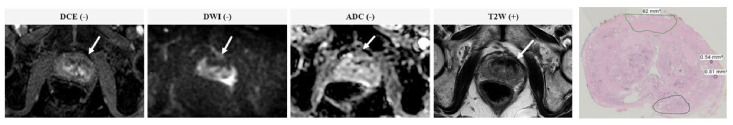
**PI-FAB 1 lesion, confirmed on histology.** PI-FAB 1 at the ablation site in the index lesion (Gleason 3 + 4, GG2) in the left anterior peripheral zone (PZ). This was confirmed on whole-mount radical prostatectomy specimen (green outline), performed for out-field recurrence at the left posterior PZ (dashed black outline).

**Figure 3:**
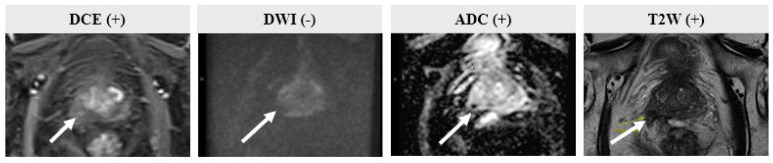
**PI-FAB 2 lesion, biopsy negative.** At 1 year post-focal therapy, mpMRI demonstrated PI-FAB 2 at the ablation site in the right PZ (index lesion Gleason 3 + 4). Targeted biopsy was negative.

**Figure 4:**
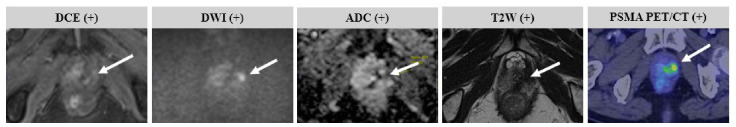
**PI-FAB 3 lesion, proven on MRI targeted biopsy.** PI-FAB 3 involving the left periurethral apex and left lateral PZ apex ablation sites (index lesion Gleason 3 + 4). Repeat biopsy was positive for clinically significant prostate cancer (recurrence Gleason 4 + 5). Corresponding PSMA imaging demonstrated tracer uptake at the ablation sites, consistent with recurrent disease.

Readers assigned a PI-FAB score of 1–3 to each ablation site. PI-FAB scores were correlated with histopathological findings from targeted biopsies at the ablation site, serving as the reference standard. PI-FAB 3 is highly suspicious for in-field recurrence and is designated a positive score. PI-FAB 2 is equivocal for recurrence. PI-FAB 1 is negative for recurrence and is designated a negative score. PI-FAB scores 1 and 2 were designated as negative, and PI-FAB 3 was designated as positive. Figure 2, Figure 3 and Figure 4 demonstrate sample images of patients with PI-FAB 1–3 lesions.

### 2.5. Evaluation of Concordance

All post-FT mpMRI and biopsy outcomes were evaluated at regular multidisciplinary team conferences comprising experienced uroradiologists, urologists, and uropathologists to determine radiological–pathological concordance. Clinically significant in-field recurrence (csPCA) is defined as the presence of ISUP/WHO (International Society of Urological Pathology/World Health Organization) GG (Grade Group) ≥ 2 identified at an ablated site on prostate biopsy. PI-FAB 3 and PI-FAB 1/2 scores are deemed true positive and true negative by the presence or absence of csPCA at ablation sites, respectively.

The diagnostic performance of each reader was evaluated using performance metrics to assess the accuracy of the PI-FAB score in predicting in-field recurrence of csPCA. In this context, in-field recurrence is defined as the presence of ISUP/WHO grade group ≥2 cancer found at a previously ablated site during a prostate biopsy. The assessment was conducted using a PI-FAB score threshold of 3; cases with a score of 3 were considered positive for recurrence.

### 2.6. Statistical Analysis

A statistical analysis was performed using R (version 4.2.2; R Foundation for Statistical Computing, Vienna, Austria). The sensitivity, specificity, positive predictive value (PPV), negative predictive value (NPV), and overall accuracy were calculated for each reader. To measure inter-rater agreement, quadratic weighted Cohen’s kappa (κ) was calculated, which evaluates the level of agreement between the two radiologists’ interpretations of post-FT mpMRI. Subanalyses were performed for each PI-FAB score category to characterize performance within each category. Overall accuracy was compared between the two independent readers.

## 3. Results

### Participant Characteristics

From May 2019 to October 2024, a total cohort of 92 patients was identified, of whom the majority underwent cryoablation (n = 89) and a small subset underwent irreversible electroporation (IRE) (n = 3). All patients had baseline pre-FT mpMRI.

The mean age was 65.5 ± 6.6 years, and the mean PSA was 7.5 ± 3.6 ng/mL. Pre-treatment ISUP/WHO Gleason grade groups were distributed as follows: grade group 1 in 3/92 patients (3.3%), grade group 2 in 63/92 patients (68.5%), grade group 3 in 20/92 patients (21.7%), grade group 4 in 6/92 patients (6.5%), and grade group 5 in 0/92 patients (0%). Baseline characteristics are summarized in Table 1. The GG1 PCa patients were excluded from analysis.

At 1 year post-FT, surveillance mpMRI with confirmatory targeted biopsy was available for 85 men. At 3 years post-FT, 32 men had paired mpMRI and confirmatory biopsy. At 5 years post-FT, seven men completed mpMRI surveillance and biopsy. A total of 140 ablation sites with confirmatory targeted biopsy were included in the analysis (Figure 1).

**Figure 5:**
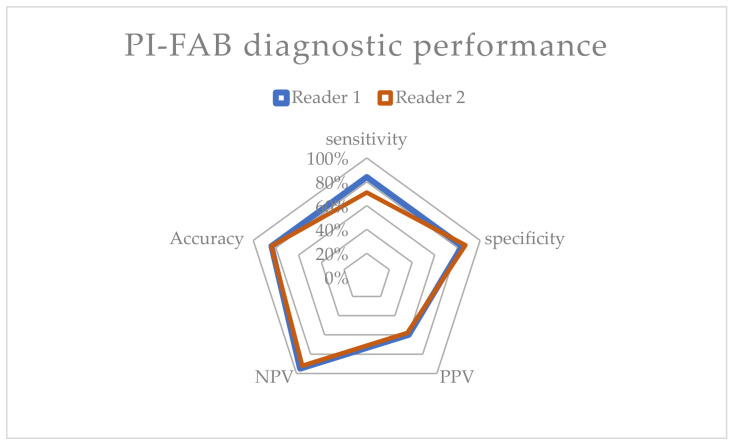
**Diagnostic performance of Reader 1 and Reader 2 (spiderweb chart).** This diagram shows the diagnostic performance of Reader 1 (blue) and Reader 2 (red), including sensitivity, specificity, positive predictive value (PPV), negative predictive value (NPV), and overall accuracy.

Diagnostic performance of post-ablation mpMRI for detecting in-field residual or recurrent prostate cancer is summarized in Table 2 and Figure 5. Reader 1 demonstrated 83.9% (95% CI: 66.3–93.4%) sensitivity and 84.4% (95% CI: 76.3–90.1%) specificity, yielding a positive predictive value (PPV) of 60.5% (95% CI: 45.9–73.3%), a negative predictive value (NPV) of 94.8% (95% CI: 88.5–97.8%), and an overall accuracy of 84.3% (95% CI: 77.4–89.3%). Reader 2 showed lower performance, with a sensitivity of 71.4% (95% CI: 52.9–84.7%), a specificity of 87.5% (95% CI: 80.1–92.3%), a PPV of 58.8% (95% CI: 42.1–73.6%), an NPV of 92.5% (95% CI: 85.8–96.2%), and an accuracy of 84.3% (95% CI: 77.4–89.3%). Overall, Reader 1 consistently demonstrated higher sensitivity and negative predictive values than Reader 2, indicating better discrimination of residual/recurrent disease on follow-up mpMRI (Figure 5).

**Figure 6:**
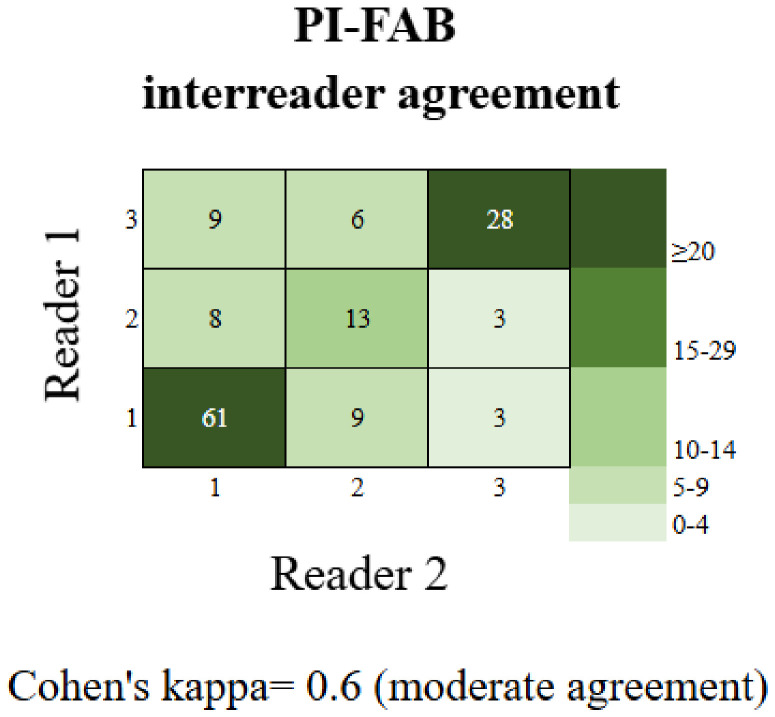
**Heatmap of PI-FAB scores by Reader 1 and Reader 2.** The heatmaps illustrate inter-reader agreement and score distributions for PI-FAB, comparing Reader 1 (y-axis) and Reader 2 (x-axis). Each cell represents the number of patients assigned a given PI-FAB score by both readers. The color gradient reflects frequency, with darker shades indicating higher levels of agreement. The calculated Cohen’s Kappa is 0.6 (moderate agreement).

Cohen’s kappa was 0.6, indicating moderate agreement between the two radiologists (Figure 6).

We analyzed diagnostic performance according to PI-FAB-stratified scores (Table 3). For PI-FAB 1 lesions, Reader 1 scored 73 lesions with 100% (95% CI: 95.0–100%) accuracy, while Reader 2 scored 78 lesions with 92.3% (95% CI: 84.2–96.3%) accuracy. For PI-FAB 2, Reader 1 scored 24 lesions with 79.2% (95% CI: 59.5–90.8%) accuracy. Reader 2 scored 28 lesions with 92.8% (95% CI: 76.5–98.2%) accuracy. For PI-FAB 3, Reader 1 scored 43 lesions with 60.5% (95% CI: 45.7–73.7%) accuracy. Reader 2 scored 34 lesions with 58.8% (95% CI: 42.2–73.7%) accuracy. Overall, these findings suggest that mpMRI interpretation is most reliable for lower PI-FAB categories, whereas higher PI-FAB scores are associated with reduced reader performance and greater interpretive uncertainty.

## 4. Discussion

### 4.1. Diagnostic Performance and Comparison with the Available Literature

This study independently validates the novel PI-FAB scoring system in the largest cohort with histopathological correlation to date. Our findings demonstrate that PI-FAB effectively detects clinically significant prostate cancer (csPCA) in-field recurrence. Diagnostic performance remained robust across readers of varying experience:

- **Reader 1 (11 years’ experience):** Sensitivity 83.9%, specificity 84.4%, PPV 60.5%, NPV 94.8%.
- **Reader 2 (4 years’ experience):** Sensitivity 71.4%, specificity 87.5%, PPV 58.8%, NPV 92.5%.
- **Overall Accuracy:** 84.3% for both readers.

Additionally, this inter-reader study found that genitourinary radiologists exhibited moderate consistency (κ = 0.6) when applying PI-FAB, suggesting moderate agreement despite differences in experience between the two readers.

The high sensitivity and specificity of the PI-FAB scoring system are particularly encouraging, as previous studies have highlighted the challenges of multiparametric magnetic resonance imaging (mpMRI) in detecting recurrence post-FT in the presence of substantial post-treatment signal changes and gland distortion [15].

Pausch et al. reported low sensitivity (14–43%) but high specificity (>95%) after focal high-intensity focused ultrasound (HIFU) [16,17], suggesting that substantial in-field recurrences were missed. Gelikman et al. reported high sensitivity (>90%) but moderate specificity (54–63%) across mixed FT modalities. A follow-up study by this group, comparing PI-FAB and TARGET scores in an extended post-HIFU cohort, found that PI-FAB scores had low sensitivity and high specificity for detecting in-field recurrences [17].

These studies highlight the trade-off between high sensitivity and relatively lower specificity. While lower specificity risks unnecessary biopsies, a highly sensitive screening tool is paramount given the investigational status of FT [18]. Furthermore, ablation modalities yield distinct tissue changes: cryoablation produces sharp, homogeneous necrosis, whereas HIFU and IRE can produce confounding cystic changes that alter early PI-FAB interpretation. Although our cohort included only three patients who had undergone IRE, we observed that cryoablation tended to create a zone of relatively homogeneous necrosis with sharper ablation margins, whereas IRE resulted in cystic changes in the treated area. It is important to note that the diagnostic performance reported in this study is primarily applicable to patients following focal cryoablation. Given the very small number of IRE patients (n = 3), these results should not be extrapolated to patients undergoing IRE, which produces distinct tissue changes and may behave differently under the PI-FAB framework. Future dedicated studies in IRE and other ablative therapy cohorts, e.g., HIFU or radiofrequency ablation (RFA), are needed to evaluate PI-FAB performance in different settings. Nevertheless, given the unknown long-term metastatic risks of untreated recurrence, prompt biopsy of suspicious in-field changes remains clinically justified [19].

Previously, we utilized PI-RADS v2.1 to evaluate post-cryoablation recurrence but noted limitations due to the lack of a post-focal therapy (FT) framework [18,20]. The recent development of the PI-FAB scoring system provided a dedicated framework which we validated in this study.

### 4.2. Granular Performance by PI-FAB Category

Stratifying performance by score reveals the operational strengths and limitations of the system:

PI-FAB 1 (Likely Benign): Both readers achieved excellent accuracy (Reader 1: 100%; Reader 2: 92%). This strong correlation with the absence of csPCA safely supports non-invasive management and avoids unnecessary biopsies.

PI-FAB 2 (Equivocal): Reader 2 outperformed Reader 1 (93% vs. 79% accuracy) when PI-FAB 2 was categorized as negative. The reversal in performance suggests significant variability in how this critical middle category is applied. This divergence implies that the imaging criteria for an “equivocal” finding are subjective and highly reader-dependent, leading to inconsistent correlations with final pathology.

PI-FAB 3 (Suspicious): Accuracy was modest for both readers (~60%), yielding a ~40% false-positive rate regardless of experience.

Consequently, while PI-FAB excels at ruling out in-field recurrence (PI-FAB 1), its ability to “rule in” disease is limited (PI-FAB 3). When encountering a PI-FAB 2 score, integrating clinical parameters such as PSA velocity and density is critical for decision-making.

### 4.3. Inter-Reader Agreement and Effect of Experience

Variability among radiologists is a known challenge in imaging, even with structured scoring systems [21]. Our two expert radiologists achieved moderate agreement (κ = 0.6) and the same overall accuracy, mirroring Gelikman et al. [15]. While Bertelli et al. reported near-perfect agreement among three radiologists of high, moderate and low experience (Gwet’s AC2 = 0.941), their cohort consisted primarily of low-risk GG1 disease (89.7%) and lacked histopathological confirmation, which may inflate agreement metrics [22]. Contemporary guidelines suggest ISUP/WHO GG1 carries very low risk of progression, metastasis, and disease-specific mortality [23,24].

The moderate interobserver agreement (κ = 0.6) highlights the challenges in evaluating ablation zones even among highly experienced uroradiologists in mpMRI interpretation. This is further compounded by the limited availability of published literature as a reference for less experienced readers in this highly challenging field of post-FT mpMRI evaluation [12].

The higher sensitivity and positive and negative predictive values achieved by Reader 1 underscore the steep learning curve of post-FT imaging. This distinction likely reflects variation in familiarity with post-treatment signal characteristics and in confidence in distinguishing expected post-procedural changes from residual or recurrent disease, consistent with prior literature [22].

Like PI-RADS [7] and PI-QUAL [25], we anticipate that inter-reader consistency will improve with structured training and wider clinical adoption [21]. These findings reflect the challenges of a novel standardized scoring system with a steep learning curve but suggest that the consistency of the PI-FAB scoring system could improve with wider use and experience. Future studies involving radiologists with diverse experience levels in a larger cohort will be crucial for validation.

It is also important to consider how image quality and acquisition protocol standardization affect diagnostic performance. In our study, all scans were acquired on 3-Tesla MRI (Magnetom Skyra or Magnetom Vida; Siemens, Erlangen, Germany) using a 60-channel pelvic phased-array coil, with a consistent multi-b-value DWI protocol (b = 0–50, 500, 1000, 1800 s/mm^2^) and DCE with gadoterate meglumine. While patient-level variability in image quality inevitably exists in a clinical dataset (due to differences in body habitus, motion, implants, or pelvic anatomy), our institutional protocol maintained a standardized acquisition framework throughout the study period. Published data on post-FT mpMRI across institutions highlight that inter-site variability in field strength, coil configuration, and contrast agent protocols can meaningfully affect signal characteristics in the ablation zone and downstream inter-reader agreement [12,14]. The absence of an endorectal coil in our protocol, while potentially reducing signal-to-noise ratio compared to some prior prostate MRI studies, reflects current standard practice in high-field imaging and was uniformly applied across readers, limiting its differential impact on inter-reader discordance. Nonetheless, inconsistencies in image quality—particularly in DCE temporal resolution and DWI b-value selection—represent an underappreciated source of PI-FAB score variability that future multicenter studies should prospectively address and report.

### 4.4. Limitations and Future Directions

There are inherent limitations to the PI-FAB scoring system. Firstly, PI-FAB was proposed by a single group based on a single institution’s clinical experience in reviewing post-FT mpMRI and through an internal audit of findings. Without a formal consensus process, its standardization may face challenges.

Secondly, PI-FAB relies heavily on DCE. The dominant sequence in PI-FAB is DCE, which may have limited application in community practices or institutions with limited hardware or expertise. While we and other experts with experience interpreting post-FT mpMRI agree that DCE is extremely important and sensitive for evaluating recurrence at ablation sites, DWI is an important adjunct, especially for recurrence in transition zones or when DCE findings are equivocal [26]. Paxton et al. also demonstrated the value of both DCE and DWI for detecting residual and recurrent cancer post-FT [27]. Importantly, T2-weighted imaging (T2-WI), while not the dominant sequence in PI-FAB, plays a critical role in characterizing ablation zone morphology. Focal T2 hypointensity that is distinct from the diffuse low signal expected with fibrosis may raise suspicion for residual tumor, particularly when corroborated by DCE and DWI findings [11]. The sequential multi-sequence approach of PI-FAB—assigning DCE the dominant role, followed by DWI/ADC and T2-WI as adjuncts—is therefore not arbitrary but reflects both the biology of post-ablation tissue and the sequence-specific signal characteristics most reliably altered by recurrent tumor.

Thirdly, treatment-related tissue changes are a major source of false-positive results with PI-FAB. Post-ablation changes, including hemosiderin deposition, dystrophic calcification, scarring, peri-ablation edema, granulation tissue, and benign inflammatory infiltrate, can produce focal enhancement on DCE, restricted diffusion on DWI, and T2 signal alterations that may mimic recurrent csPCA [11,12]. These changes are most pronounced in the early post-treatment period but may persist beyond 12 months, underscoring the diagnostic challenge at the 1-year surveillance time point used in our study. In our dataset, approximately 40% of PI-FAB 3 lesions were confirmed negative on targeted biopsy by both readers, reflecting a false-positive rate inherent to imaging in an ablated prostate. PI-FAB addresses this challenge by incorporating pre-treatment imaging appearance and ablation-site context into the scoring rubric, thereby distinguishing expected post-ablation change from suspicious enhancement patterns [11]. Nonetheless, recognizing the imaging spectrum of treatment-related benign change remains a critical component of reader training and is an area where structured post-FT reporting education and reference atlases would add significant value. Lastly, PI-FAB relies on pre-treatment data which may not be available at the time of reporting, especially in specialized tertiary referral centers where prior treatment was carried out off-site. In our study which arises from a urology–radiology–pathology integrated focal therapy program, pretreatment mpMRI and prior ablative treatment information were readily available to the readers.

Similarly, our study has a few limitations. First, it is a single-institution retrospective study. Further refinement to the scoring system ideally requires larger, multicenter, prospective validation studies to achieve international consensus. Second, the optimal follow-up time points for post-FT MRI surveillance and biopsy also remain poorly established. The PI-FAB proposal has not made specific recommendations on surveillance mpMRI interval, and it is known that MRI findings continue to evolve after FT [28]. In our study, all patients who underwent focal therapy had the first surveillance mpMRI 12 months post-FT, followed by a mandatory targeted and systematic prostate biopsy. Subsequently, we analyzed biopsies independently of the post-FT time interval to maximize sample size.

Nonetheless, our study validates the PI-FAB score in the largest sample size to date, with the longest follow-up time, with paired mpMRI and histopathological results from targeted prostate biopsies following each mpMRI as the reference standard.

## 5. Conclusions

The PI-FAB scoring system demonstrated robust diagnostic performance for detecting residual cancer or in-field recurrence at ablation sites after focal cryoablation, with particular strength in excluding local recurrence. Importantly, the diagnostic metrics reported here are derived from the largest histopathologically confirmed cohort in the PI-FAB validation literature to date, spanning 140 lesion sites over up to 5 years of follow-up. The results are primarily applicable to cryoablation; given the very small number of IRE patients (n = 3) in this cohort, extrapolation to IRE should be made with caution until dedicated IRE-specific studies are conducted. Despite differences in experience level between the two genitourinary radiologists, moderate inter-reader agreement was achieved, with the more experienced reader demonstrating higher sensitivity and predictive values. Together, these findings provide meaningful evidence supporting the validity and potential clinical utility of PI-FAB as a standardized post-focal therapy mpMRI surveillance tool. Further multicenter prospective studies encompassing a broader range of reader experience levels and ablative modalities will build on this foundation.

## Figures and Tables

**Table 1:** **Baseline patient demographics and disease characteristics.** Patients’ age, ethnicity, pre-treatment serum prostate-specific antigen (PSA) level, and pre-treatment ISUP/WHO grade group.

**Total number of patients**	**n = 92**
**Age, mean (SD)**	65.5 (6.6)
**Ethnicity**	
Chinese	84 (91.3%)
Malay	2 (2.2%)
Indian	1 (1.1%)
Others	5 (5.4%)
Pre-treatment Serum PSA (ng/mL), mean (SD)	7.5 (3.6)
**Pre-treatment ISUP/WHO grade group (GG), n (%)**	
GG1	3 (3.3%)
GG2	63 (68.5%)
GG3	20 (21.7%)
GG4	6 (6.5%)
GG5	0 (0)

**Table 2:** **Diagnostic performance of Reader 1 and Reader 2.** Sensitivity, specificity, positive predictive value (PPV), negative predictive value (NPV), and overall accuracy were calculated.

	Reader 1	Reader 2
**Sensitivity (95%CI)**	83.9% (66.3–93.4%)	71.4% (52.9–84.7%)
**Specificity (95%CI)**	84.4% (76.3–90.1%)	87.5% (80.1–92.3%)
**PPV (95%CI)**	60.5% (45.9–73.3%)	58.8% (42.1–73.6%)
**NPV (95%CI)**	94.8% (88.5–97.8%)	92.5% (85.8–96.2%)
**Accuracy (95%CI)**	84.3% (77.4–89.3%)	84.3% (77.4–89.3%)

**Table 3:** **Overall accuracy for Reader 1 and Reader 2, stratified by PI-FAB score**. Scores are presented as x/y (%), where “x” denotes the number of correctly classified lesions and “y” denotes the total number of lesions assigned to that PI-FAB score.

**Overall Accuracy Stratified by Score, Reader 1 vs. Reader 2**			
**PI-FAB score**	**1**	**2**	**3**
**Reader 1 (95%CI)**	73/73 = 100% (95.0–100%)	19/24 = 79.2% (59.5–90.8%)	26/43 = 60.5% (45.7–73.7%)
**Reader 2 (95%CI)**	72/78 = 92.3% (84.2–96.3%)	26/28 = 92.8% (76.5–98.2%)	20/34 = 58.8% (42.2–73.7%)

## Data Availability

The original contributions presented in this study are included in the article. Further inquiries can be directed to the corresponding author.

## Abbreviations

The following abbreviations are used in this manuscript, sorted in alphabetical order:

ADC	Apparent Diffusion Coefficient
csPCA	Clinically Significant Prostate Cancer
DCE	Dynamic Contrast-Enhanced
DWI	Diffusion Weighted Imaging
FT	Focal Therapy
GG	Grade Group
HIFU	High-Intensity Focused Ultrasound
IRB	Institutional Review Board
IRE	Irreversible Electroporation
ISUP/WHO	International Society of Urological Pathology/World Health Organization
mpMRI	Multiparametric Magnetic Resonance Imaging
MRI-TRUS	Magnetic Resonance Imaging–Transrectal Ultrasound
NPV	Negative Predictive Value
PI-FAB	Prostate Imaging After Focal Ablation
PI-RADS	Standardized Prostate Imaging: Reporting and Data System
PPV	Positive Predictive Value
PSA	Prostate-Specific Antigen
PSMA-PET	Prostate-Specific Membrane Antigen Positron Emission Tomography
PZ	Peripheral Zone
T1W	T1 Weighted Imaging
T2W	T2 Weighted Imaging
TZ	Transitional Zone
